# Systematic Review of the Long-Term Effects of Transgender Hormone Therapy on Bone Markers and Bone Mineral Density and Their Potential Effects in Implant Therapy

**DOI:** 10.3390/jcm8060784

**Published:** 2019-06-01

**Authors:** Rafael Delgado-Ruiz, Patricia Swanson, Georgios Romanos

**Affiliations:** 1 Prosthodontics and Digital Technology, School of Dental Medicine, Stony Brook University, Stony Brook, NY 11794, USA; patricia.swanson@stonybrookmedicine.edu; 2 Periodontology, School of Dental Medicine, Stony Brook University, Stony Brook, NY 11794, USA; georgios.romanos@stonybrookmedicine.edu

**Keywords:** transgender, long-term pharmacotherapy, bone markers, bone mineral density, implants

## Abstract

This study seeks to evaluate the long-term effects of pharmacologic therapy on the bone markers and bone mineral density of transgender patients and to provide a basis for understanding its potential implications on therapies involving implant procedures. Following the referred Reporting Items for Systematic Reviews and Meta-Analyses (PRISMA) guidelines and well-defined PICOT (Problem/Patient/Population, Intervention, Comparison, Outcome, Time) questionnaires, a literature search was completed for articles in English language, with more than a 3 year follow-up reporting the long-term effects of the cross-sex pharmacotherapy on the bones of adult transgender patients. Transgender demographics, time under treatment, and treatment received were recorded. In addition, bone marker levels (calcium, phosphate, alkaline phosphatase, and osteocalcin), bone mineral density (BMD), and bone turnover markers (Serum Procollagen type I N-Terminal pro-peptide (PINP), and Serum Collagen type I crosslinked C-telopeptide (CTX)) before and after the treatment were also recorded. The considerable variability between studies did not allow a meta-analysis. All the studies were completed in European countries. Transwomen (921 men to female) were more frequent than transmen (719 female to male). Transwomen’s treatments were based in antiandrogens, estrogens, new drugs, and sex reassignment surgery, meanwhile transmen’s surgeries were based in the administration of several forms of testosterone and sex reassignment. Calcium, phosphate, alkaline phosphatase, and osteocalcin levels remained stable. PINP increased in transwomen and transmen meanwhile, CTX showed contradictory values in transwomen and transmen. Finally, reduced BMD was observed in transwomen patients receiving long-term cross-sex pharmacotherapy. Considering the limitations of this systematic review, it was concluded that long-term cross-sex pharmacotherapy for transwomen and transmen transgender patients does not alter the calcium, phosphate, alkaline phosphatase, and osteocalcin levels, and will slightly increase the bone formation in both transwomen and transmen patients. Furthermore, long-term pharmacotherapy reduces the BMD in transwomen patients.

## 1. Introduction

The term “transgender” describes a population experiencing incongruence between their physical sex characteristics (assigned gender) and their gender identity (the extent to which people experience themselves to be like others of one gender) [1]. In some instances, as a result of the incongruence between assigned gender and gender identity, an individual can suffer distress (gender dysphoria), which may be accompanied by physical or mental health issues [2].

Patients experiencing gender dysphoria present with different requests; these patients include subjects requiring a transition to the opposite sex with the support of different medical specialists, those who aspire to live outside stereotypic roles without biological transformation, individuals who need medical care for health issues other than gender, and patients who require psychiatric support [3]. Those subjects who wish to transition to the opposite sex usually receive pharmacotherapy to increase their secondary sexual characteristics and may also undergo surgical procedures for gender reassignment [4].

The pharmacotherapy for sex transition is based on hormonal and non-hormonal treatments with the goal of partial or full inhibition of the patient’s gonadal axis and an increase in their secondary sex characteristics [5,6]. Male-to-female patients utilize hormonal estrogen preparations (different compounds of estradiol) and anti-androgens, such as spironolactone and gonadotropin-releasing hormone agonists (GnRH) [7]. Spironolactone decreases the response to androgens because it inhibits the androgen receptor, possesses a feminizing effect, reduces facial hair, and slows male alopecia. Furthermore, it provides additive effects to estrogen intake, allowing the estrogen dosage to be reduced (thus reducing the risks involved with higher doses of estrogen) [8]. On the other hand, GnRH agonists inhibit testosterone secretion by restraining luteinizing hormone secretion and thereby result in gonadal suppression [7,8].

Female-to-male patients utilize testosterone-based and non-testosterone-based therapy [9]. Non-testosterone therapies include medroxyprogesterone, GnRH agonists, 5-alpha reductase inhibitors, and selective serotonin receptor inhibitors (SSRIs) [10]. These therapies can support the offset of the side effects of testosterone therapy and support other aspects of the sex transition. For example, medroxyprogesterone assists with menstrual cessation, the GnRH agonists also reduce menses and refractory uterine bleedings, as well as the 5-alpha reductase inhibitors, and SSRI’s help with hair overgrowth or hair loss [9,10,11].

However, as a consequence of the pharmacological treatment received by the patients who have transitioned or are in the process of transitioning to the opposite sex, unintended systemic biological changes may occur. These may include an increased cardiovascular risk in both transwomen and transmen [4], a significant increase of body mass index and systolic and diastolic blood pressure in transmen [12], and osteoporosis at the lumbar spine and distal arm in transwoman [13]. Reported adverse effects also include venous thromboembolism, fractures, cardiovascular disease, stroke, and hormone-dependent cancers [14], and finally, with some disagreement, osteoporotic changes in the bone mass, density, and geometry in transmen and transwomen patients have been described [15].

Several investigations confirmed that testosterone and estrogens are essential to control bone health in men and women [16,17,18]. In men, testosterone plays a vital function for skeletal equilibrium, and estradiol is required for skeletal development [17]. In women, estrogens participate in bone homeostasis, but the effect of androgens is less clear [16,18]. This insinuates that bone metabolism might suffer variations as a result of transgender hormone therapy.

During hormone therapy, bone changes are evaluated periodically through bone mineral density (BMD), and bone turnover marker (BTM) analysis. BMD is estimated with Dual X-ray Absorptiometry (DEXA) and for the evaluation of active bone remodeling, BTMs are evaluated in serum and/or urine [19]. This allows for the prediction of the risk of osteoporosis, and the monitoring of treatment progression (effects of the therapy on bone metabolism and structure) [20].

The World Health Organization (WHO) standards consider that osteoporosis is present, when the T-score is <2.5 (standard deviation contrasted to the mean value of BMD of the specific population target) [21]. The BTMs for bone formation obtained from serum are: osteocalcin (OC), total alkaline phosphatase (ALP), bone-specific alkaline phosphatase (bone ALP), procollagen type I C-terminal pro-peptide (P1CP), and procollagen type I N-terminal pro-peptide (P1NP) [22].

Meanwhile, the BTMs for bone resorption can be obtained from serum (collagen type I cross-linked C-telopeptide (sCTX), carboxyterminal telopeptide of type I collagen (ICTP), tartrate-resistant acid phosphatase (TRACP), tartrate-resistant acid phosphatase 5b (s-TRACP 5b), and urine (collagen type I crosslinked N-telopeptide (uNTX)), collagen type I cross-linked C-telopeptide (uCTX), total pyridoline (uPYD), and total deoxypyridoline (uDPD) [23].

Nearly 0.6% of U.S. adults identify themselves as transgender [24], and global transgender prevalence has been reported in the United Kingdom (0.5%) [25], Belgium (0.6%) [26], and the Netherlands (0.9%) [27]. It has been reported that the transgender population encounters difficulties regarding access to health care [28] and that healthcare providers are not suitably prepared to adequately serve the transgender community [28].

Focusing on the oral care for transgender patients, the literature in the field is very scarce and mainly centered around dental fear [29] and dental education related to the topic [30,31,32], with only one clinical case published that related to full mouth rehabilitation of a transgender patient [33]. Unfortunately, there are no references to the bone parameters of the transgender population linked to oral and maxillofacial surgery or implant procedures, and, as Ludwig et al. (2018) stated, “We cannot provide evidence-based dental care to a subset of the population, if that population has yet to be studied.” [34].

Pharmacologic therapy may influence the bone structure of transgender patients receiving or rehabilitated with titanium implants or having oral and maxillofacial surgical procedures. Furthermore, the long-term effects of these therapies on bone mineral density, bone metabolism, and bone resorption are unknown.

Therefore, the goals of this systematic review were twofold:

- First, to answer the following PICOT (Problem/Patient/Population, Intervention, Comparison, Outcome, Time) question: In adult transgender patients (transwoman and transmen), receiving long-term pharmacologic therapy, are the bone markers and bone mineral density affected differently?
- Second, to provide a theoretical basis for a better understanding of the implications of the long-term pharmacologic therapy in the adult transgender patient on therapies involving orthopedic or dental implants.

## 2. Materials and Methods

The Preferred Reporting Items for Systematic Reviews and Meta-Analyses (PRISMA) guidelines were followed [35]. Electronic and manual searches were completed in Medline, EMBASE, and PubMed from January 2018 to December 2018 for the following search terms: “transgender AND bone health” OR “transgender AND cortical bone” OR “ transgender AND trabecular bone” OR “transgender AND bone structure” OR “transgender AND bone metabolism” OR “transgender AND bone mineral density” OR “transgender AND bone loss” OR “cross-sex hormone AND bone health” OR “pharmacotherapy AND transgender” OR “cross-sex AND cortical bone” OR “cross-sex AND trabecular bone” OR “cross-sex AND bone structure” OR “cross-sex AND bone metabolism” OR “cross-sex AND bone mineral density” OR “cross-sex AND bone loss” OR “cross-sex AND bone health”.

An additional manual search was completed within the references provided in the included manuscripts to identify other reports not returned by the electronic searches.

### 2.1. Study Inclusion Criteria

Long-term clinical studies completed in adult transgender populations (transwomen and transmen) that received pharmacological treatment were included as per the following inclusion criteria:

- Papers published in the English language from January 1990 through to December 2018;
- Publications reporting clinical studies with three or more years follow-up;
- Publications reporting the biological effects of cross-sex pharmacologic therapy in the bone markers, bone metabolism, and bone mineral density of transgender patients;
- Papers reporting the pharmacologic therapy used for transgender adults (including both retrospective and prospective studies).

If a study appeared duplicated, just the study that featured the most extensive follow-up, or the most recent study, was included.

### 2.2. Study Exclusion Criteria

- Articles published in languages other than English;
- Publications reporting less than three years of follow-up;
- Publications detailing the effects of cross-sex pharmacologic therapy in teenagers or young transgender patients;
- Publications reporting effects of cross-sex pharmacologic therapy not including the effects on bone
- Animal studies and in-vitro studies;
- Other systematic reviews and meta-analyses;
- Duplicated studies;
- Case reports.

Two reviewers (R.D. and G.R.) completed initial independent searches using the search terms. After the initial search, the titles and abstracts of the returned articles were read and articles that satisfied the inclusion criteria were selected. Afterward, the included articles were read in full and evaluated for final eligibility. The discrepancies between the reviewers were solved with the participation of a third, blinded reviewer (P.S.).

### 2.3. Data Extraction

The following information was extracted from all the included manuscripts:

- Transgender demographics: Number of males to female (transwomen) or female to male (transmen) patients included in each study;
- The duration of hormone-therapy treatment expressed in years or months;
- Type of hormone received by the patient and dosage.

#### 2.3.1. Primary Outcomes

- Changes in bone metabolism marker levels calcium (mmol/L), phosphate (mmol/L), alkaline phosphatase (U/L), and osteocalcin (µg/L). Changes were measured at baseline and after treatment or at different time points for each of the bone metabolism markers.
- Changes in the BTMs including Serum Procollagen type I N-Terminal pro-peptide (PINP) (ng/mL) for evaluation of the bone formation, and Serum Collagen type I cross-linked C-telopeptide (CTX) (ng/mL) for evaluation of bone resorption. Changes were recorded at baseline and at different time points.
- Changes in the BMD. BMD values were registered at baseline and after the completion of treatment or at baseline and at different time points.

The obtained data were organized in tables ordered by year of publication from the oldest to the newest publication.

#### 2.3.2. Risk of Bias

To determine the risk of bias in the included studies, the risk of bias assessment tool (RoB 2 tool) [36] was used. Five elements (domains) were assessed for each included manuscript including the randomization process, deviations from intended interventions, missing outcome data, measurement of the outcome, and selection of the reported result. Each domain was graded, and the risk of bias was scored as low risk of bias, some concerns, or high risk of bias, following the descriptors of the RoB 2 tool [36].

### 2.4. Statistical Analysis

Descriptive statistics, percentages, mean and standard deviations, and forest plot graphics were used for the presentation of the data. Meta-analysis was completed, if applicable, using a random effects model. Meta-analysis software (Comprehensive-Meta-analysis 3.0, Biostat, NJ, USA) was used for the statistical comparisons.

## 3. Results

**Figure 1:**
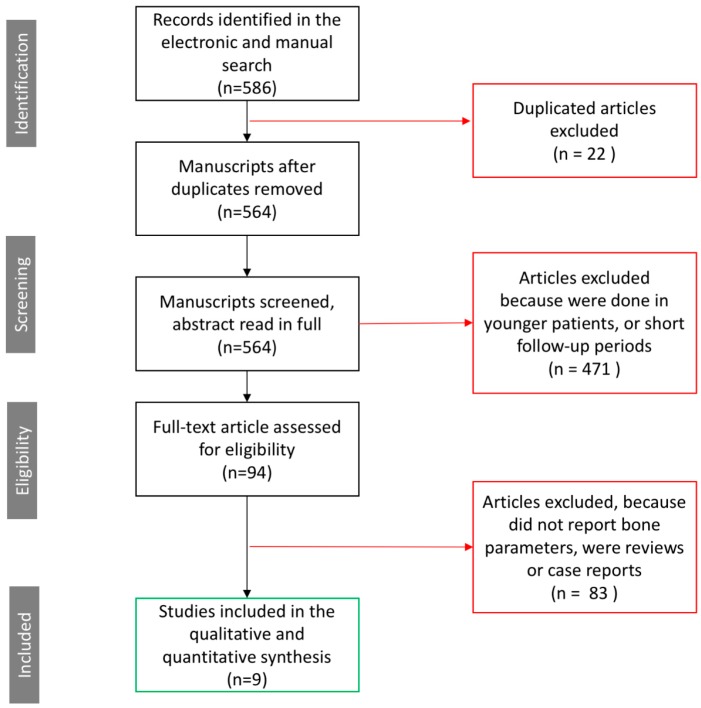
The Preferred Reporting Items for Systematic Reviews and Meta-Analyses (PRISMA) workflow. From the initial 586 articles, only nine articles fulfilled the inclusion criteria and were considered for this systematic review.

The initial search returned 564 articles. After reading the titles and abstracts, 471 articles were excluded. The remaining 93 articles were read in full and 84 articles were excluded (according to the exclusion criteria). Finally, nine manuscripts that fulfilled the inclusion criteria were included for this review (Figure 1).

### 3.1. Transgender Demographics and Time under Treatment

Nine long-term studies featuring subjects who had undergone more than three years of pharmacotherapy treatment were included. Three studies were featured only male (M) to female (F) transgender patients, two studies were only included F to M transgender patients, and four studies featured both groups (M to F and F to M) [37,38,39,40,41,42,43,44,45]. In addition to the pharmacotherapy, all the patients received sex reassignment surgery. The shortest period of evaluation was 3.5 years [40], and the longest was 18 years [45]. The total population studied was 1640 patients (921 M to F and 719 F to M transgender patients) (Table 1).

### 3.2. Type of Hormone Received and Dosage

Different treatment modalities and different dosages were received by the patients. No comparison was possible between studies based on the used drugs. Variations between hormone type/dose/time as well as variations to the treatment due to adjustments by the provider and the individual patient’s characteristics precluded the comparisons.

• M to F

1. Cyproterone + estrogens (high doses) [37].
2. Ethinyl estradiol + cyproterone acetate and oral estrogens and estradiol valerate [38].
3. Cyproterone acetate 2 mg/day + ethinyl estradiol 35–100 µg/day, after sex reassignment surgery estradiol valerate or 17-beta estradiol 2–4 mg/day [39].
4. Cyproterone acetate 50–100 mg/day + ethinyl estradiol 25–50 μg/day, after surgery ethinyl estradiol 25–50 μg/day or estradiol valerate 2 mg/day or conjugated equine estrogens 1.25 mg/day.
5. or transdermal estradiol [40].
6. Cyproterone acetate 50–100 mg/day/1 year + exogenous estrogen, after surgery all received estrogens [41].
7. Estrogens and anti-androgens until gonadectomy [42].

• F to M

Several forms of testosterone are administered

1. Testosterone esters 250 mg/IM every 3 weeks, before and after surgery [38].
2. Testosterone decanoate 100 mg, or testosterone isocaproate/fenylpropionate 60 mg, or testosterone propionate 30 mg/mL 2–3 weeks, or testosterone undecanoate 1000 mg 12 weeks, or transdermal testosterone 50 mg daily, or testosterone undecanoate 40 mg/day + testosterone gel 50 mg per 5 g, 50 mg daily [41].
3. Testosterone [42].
4. Different testosterone compounds [43].
5. Testosterone isobutyrate, and after surgery, testosterone isobutyrate 25 mg intramuscular every week, or testosterone propionate 250 mg every 3 weeks intramuscular, or testosterone undecanoate 40 mg/4 times/day [45].

### 3.3. Bone Metabolism Marker Levels Before and After the Treatment

Calcium, phosphate, alkaline phosphatase, and osteocalcin were used in three of the studies. Data could not be compared due to the differences in population and methods of detection. In addition, the measuring units varied between mg/dL and mmol/L (Table 2).

### 3.4. The Two Main Bone Turnover Markers

Both markers (PINP and CTX) were evaluated in four studies [40,41,42,43]. Meanwhile, CTX alone was evaluated in one study [45] (Table 3).

### 3.5. Bone Mineral Density (BMD), Method of Evaluation and Anatomical Areas Evaluated

The BMD was evaluated at different anatomical locations: lumbar spine (100%), femoral neck (60%), and less frequently at the total hip, distal forearm, or the whole body [37,38,39,40,41,42,43,44,45].

The method for the evaluation of the BMD was dual-energy X-ray absorptiometry (DXA) (90%). In addition, peripheral quantitative computed tomography (pQCT) was used for the evaluation of the bone architecture (10%) [40,41] (Table 1).

**Figure 2:**
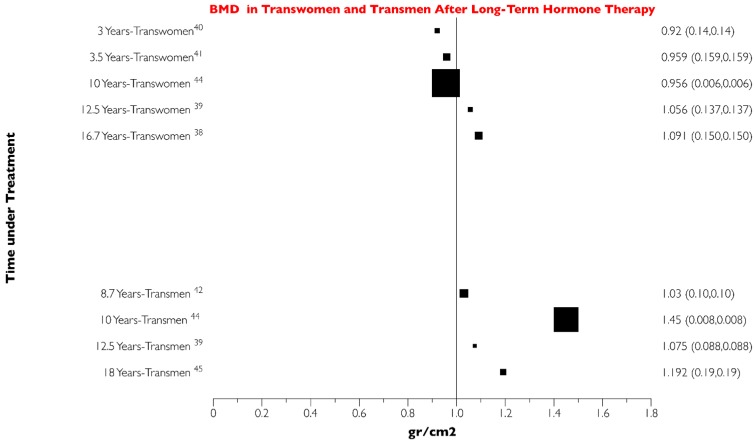
Evolution of the mean global bone mineral density (BMD) in transwomen (M to F) over a period of 17 years. Evolution of the mean global bone mineral density (BMD) in transmen (F to M) over a period of 18 years. It can be appreciated the trend toward lower BMD in transwomen compared to transmen.

When evaluating the BMD variations related to the time of pharmacotherapy in transwomen (M to F), the range was about 0.4 gr/cm^2^ over a 14 year period. Meanwhile, the BMD variation in transmen (F to M) was in the range of 0.6 gr/cm^2^ over a nine year period (Figure 2).

The BMD value for the longest evaluation period for transwomen (17 year follow-up) was 1.08 gr/cm^2^ and for transmen was 1.19 gr/cm^2^ (18 years follow-up).

### 3.6. Risk of Bias Assessment

**Figure 3:**
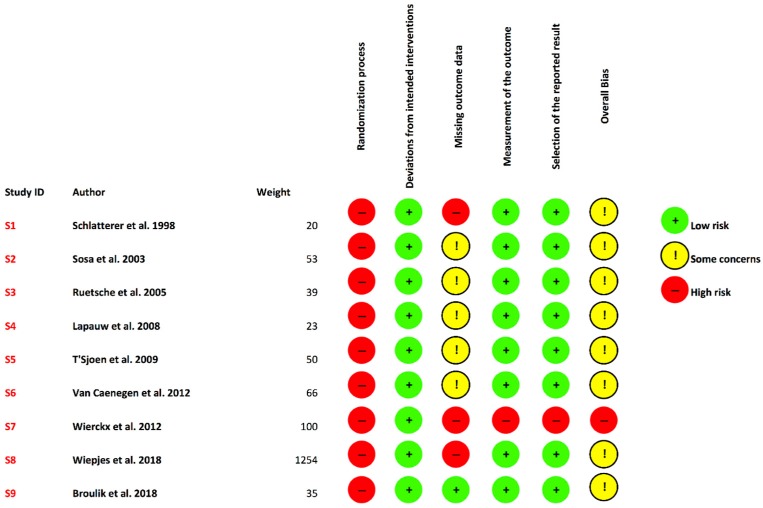
Risk of bias assessment. From the nine included studies, eight had some concerns and only one study showed an overall high risk of bias. S (study identification), Weight (sample size/number of patients per study).

None of the nine studies were randomized (high risk of bias), there were no deviations from the intended interventions (low risk of bias), there were eight studies missing at least one of the outcomes’ data (high risk of bias), when measured, the outcomes were properly assessed (low risk of bias), and there were no problems with the selection of the reported result (low risk of bias). Overall, there were some concerns related to missing information on outcomes and the lack of randomization (Figure 3).

## 4. Discussion

The purpose of this systematic review was to evaluate the bone markers and BMD of transwomen (M to F) and transmen (F to M) patients after long-term pharmacotherapy treatment for feminization or virilization with or without sex reassignment surgery.

The obtained information might provide the clinicians with a reference for the bone characteristics of transgender patients receiving long-term hormone therapy and a baseline for studying the future implant site and the peri-implant bone characteristics in this patient population.

There were very few long-term studies (greater than 3 years) [37,38,39,40,41,42,43,44,45], with substantial group variability in age, drug and dosage, time under treatment, and biochemical markers for bone metabolism that precluded the statistical comparisons. Hence, the data was represented as percentages, mean, and standard deviations when feasible.

### 4.1. Transgender Demographics and Time under Treatment

These data were gathered to understand the trends of the transgender population under pharmacotherapy supplementation. The results showed that the demographics of adult transgender M to F and F to M populations were comparable and the long-term follow-up studies were 100% based on European populations. The longest reported follow-up period extended up to 18 years [45].

### 4.2. Type of Pharmacotherapy Received and Dosage

Transgender women (M to F) before and after gonadectomy received different forms of estrogens and testosterone suppressors (cyproterone acetate and spironolactone); meanwhile, transgender men (F to M) received mainly testosterone therapy before and after surgery [37,38,39,40,41,42,43,44,45].

Different dosages and various methods of administration (intraoral, sublingual, intramuscular, patches, implants, and subcutaneous injection) were observed. The differences in pharmacologic treatment were the result of patient-centered approaches more than those that were guided by the specific pharmacological protocol from the treatment center [37,38,39,40,41,42,43,44,45].

### 4.3. Bone Metabolism Markers and Their Potential Effect on Implant Therapy

Calcium and phosphates are responsible for calcium homeostasis and participate in the acid–base balance and also facilitate the release of growth factors embedded in bone [38]. The action of the osteoclasts on the calcified bone matrix facilitates its dissolution and releases calcium ions into the blood to form blood calcium [46]. In a parallel action, the calcium contained in the blood flow can be deposited onto the bone to form bone calcium, mediated by the osteoblasts. These phenomena are regulated by enzymes and hormones (vitamin D, calcitonin, parathyroid hormone, and other metabolites) [47].

In the presence of metabolic imbalances produced by the cross-sex pharmacologic therapies, the blood calcium balance system can be altered, thereby playing an important role as an ethiological factor for pharmacologically induced osteoporosis [48].

These pharmacologic therapies can also alter the phosphorus/calcium ratio. The reduction of phosphorus can alter the calcium absorption, while increased phosphorus concentrations can increase the oxidative stress as well as the hormonal balance between phosphates, calcium, and vitamin D. This might be conducive to adverse effects on mineral metabolism and increased bone loss [49,50].

Alkaline phosphatase and osteocalcin are both bone formation markers [50,51]. Bone alkaline phosphatase (ALP) regulates bone mineralization [51]. There are also liver ALP isoforms that differ only by posttranslational modifications. However, the immunoassays with monoclonal antibodies better recognize bone isoforms [52]. Meanwhile, osteocalcin is a major non-collagenous protein synthesized by osteoblasts and odontoblasts, and its circulating levels are highly specific for bone formation. It is degraded and excreted by the kidneys [53].

Calcium and phosphate are an essential part of bone metabolism, and their depletion can result in reduced bone mineral density, changes in the bone structure (increased trabecular spacing and reduction of cortical bone thickness), osteoporosis, and delayed osseointegration [54]. Not all osteoporotic signs are the result of calcium, phosphate, or vitamin D deficiency, but these are important factors associated with optimal bone health [55,56]. Their depletion can also result from low intake, vitamin D deficiency, and changes in the metabolism induced by disease or medications [57,58].

The results of the present review showed that calcium and phosphate levels, as well as alkaline phosphatase and osteocalcin, remained within similar values after the long-term pharmacotherapy for transgender M to F and F to M patients, thereby demonstrating that the administered therapies had minimal effects on calcium/phosphate balance and alkaline phosphatase and osteocalcin levels [37,45].

### 4.4. Two Main Bone Turnover Markers

Following the recommendations by the International Osteoporosis Foundation (IOF) and the International Federation of Clinical Chemistry (IFCC) for the quantification of the bone turnover (bone resorption and bone formation processes), the N-terminal pro-peptide of type I procollagen (PINP) and C-telopeptide of type I collagen (CTX-I) were recorded [59]. Respectively, PINP measures bone formation and CTX-I measures bone resorption [59].

This review showed that hormone therapy induced an increase in the PINP values in M to F patients (17 ng/mL to 23 ng/mL) and an increase of PINP in F to M patients (>10 ng/mL) [37,38,39,40,41,42,43,44,45]. Meanwhile, the CTX values in M to F and F to M were inconsistent [37,38,39,40,41,42,43,44,45].

During transgender hormone therapy, the goal is similar to the goal of the antiresorptive treatment—to lower the PINP by at least 10 ng/mL and <35 ng/mL during bone resorption. Meanwhile, during bone formation, the goal is to raise the PINP by at least 10 ng/mL to achieve a level of >69 ng/mL [60].

Therefore, it seems that the long-term administered pharmacotherapy for M to F and F to M transgender patients can produce a slight increase in the bone formation rates evaluated with the PINP [40,41,42,43]. It should also be considered that these PINP values can change over time, induced by factors such as age, metabolism changes, and non-compliant treatment interruption [37,38,39,40,41,42,43,44,45].

### 4.5. Bone Mineral Density (BMD)

The BMD condition of M to F and F to M transgender patients receiving long-term cross-sex pharmacotherapy is contradictory. For Sosa et al. (2003) [38], Ruetsche et al. (2005) [39], Van Caenegem et al. 2012 [42], and Wiepjes et al. 2018 [44], both M to F and F to M patients will possess a stable or increased BMD compared to matched male or female controls.

The authors explain their findings based on the protective effects of estrogens in M to F patients against bone resorption, mediated by increased serum levels of estradiol [38], mediated by IGF1 (insulin-like growth factor) [39]. Meanwhile, in F to M patients, the preservation or increase of the BMD could be produced by the long-term effects of testosterone, reduced estrogen levels, and a muscle mass increase, which, all together, might result in reduced resorption rates [38,39,42,44].

On the other hand, according to Schlatterer et al. (1998) [37], Lapauw et al. (2007) [40], T’Sjoen et al. (2009) [41], and Wierckx et al. (2012) [43], the BMD of M to F patients was reduced, and signs of osteoporosis of the lumbar spine and distal arm were observed, but F to M patients did not show reduced BMD. Apparently, the effects of muscle mass reduction, non-compliance to the treatment (which can result in androgen deficiency), inadequate estrogen dosage, and sedentary lifestyles produced the bone resorption experienced by the M to F transgender patients [37,39,40,41,43] (Table 1).

#### 4.5.1. BMD Changes and their Potential Relationship to Dental Implants

The local properties of the future implant bed (bone mineral density and bone structure), as well as the primary implant fixation (primary stability), are essential factors that can reduce micromotion and will allow immediate loading protocols [61,62]. Moreover, bone quality, quantity, implant geometry, and surgical technique have been considered factors that can influence the presence of micromotion or the presence of implant stability [63]. When the bone mineral density is low, the primary implant stability cannot be achieved unless certain modifications are completed during the implant bed preparation, for example—the use of specific implant designs (tapered, self-tapping) [64], under-drilling, bone condensation, or osseo-densification techniques [65].

There is also a link between low bone density, osteoporosis, and implant failure [66] and low bone density and lower osseointegration [67]. However, recent systematic reviews showed that the implant survival rates and marginal bone loss of unloaded implants were similar in implants inserted in low-density bone compared to implants inserted in normal-density bone [68,69]. Furthermore, it was reported that low bone mineral density values found in a group of patients with osteoporosis and osteopenia did not influence the implant osseointegration after 24 months of follow-up [70]. However, when the implant requires higher primary stability (i.e., immediate loading protocols, single-body implants), the bone mineral density is a factor that should be considered before the load protocols are applied [71]. Marquezan et al. demonstrated that there is a direct association between BMD and primary implant stability (as the bone density increase, the primary stability increase). [71].

#### 4.5.2. Hypothesis for the Effects of Hormone Therapy for Transgender Patients on BMD and Its Potential Relation to Dental Implants

It seems that there is some risk of reduced BMD in M to F transgender patients receiving long-term cross-sex pharmacotherapy. Therefore, when performing dental implant procedures in such patients, the precautions followed in osteoporotic patients should be considered [66,67,68,69]. In addition, based on the bone changes observed in the present study over the long-term follow-up, it seems reasonable to monitor bone parameters before procedures involving dental implants in transgender patients [72]. Finally, when considering the risk factors for long-term implant survival (diabetes mellitus, age, smoking, and immediate loading), the BMD was the most critical factor determining implant survival (lower BMD values resulted in lower implant survival rates) [73].

### 4.6. Strengths and Limitations of the Present Work

The strengths of the present paper are that this is the first systematic review that has compiled the effects of the cross-sex pharmacotherapy on bone metabolism markers, BTMs, and BMD. Additionally, strict inclusion and exclusion criteria and adequate calibration were followed by the investigators involved in the data collection and data analysis. Finally, a hypothesis for the potential effects of long-term hormone therapy on dental implant therapy was provided.

There are limitations to the present work—firstly, the low number of studies included and the preclusion of any statistical comparisons; secondly, the exclusion of the effects of short-term (less than 3 years) cross-sex pharmacotherapy and their effects in younger patients. Moreover, the multiple variables that, at a certain point, can affect the bone metabolism and structural characteristics of this population (sex-reassignment surgery and aging) further limit the comparison of treatments.

### 4.7. Recommended Future Steps

Given the lack of information regarding the effects of hormone therapy in transgender patients on bone healing, implant osseointegration, peri-implant health, and implant survival, clinical studies compiling such information are recommended.

## 5. Conclusions

Within the limitations of this systematic review, the following conclusions can be drawn:

- Long-term pharmacotherapy for transgender patients does not alter the calcium, phosphate, alkaline phosphatase, and osteocalcin bone markers.
- Long-term pharmacotherapy for transgender patients will slightly increase the bone formation, expressed with increased PINP turnover markers.
- Long-term cross-sex pharmacotherapy for M to F transgender patients will produce a slight reduction in bone mineral density.

## Figures and Tables

**Table 1:** Demographics, time receiving treatment, and medication received. Bone mineral density (BMD) and method of evaluation and study conclusions related to bone structure, bone metabolism, and bone mineral density. Variability was observed in drug dosages and regions for BMD. M, male; F, female.

Author, Year of Publication, Location	Sample		Time Receiving the Treatment	Hormone and Dosage		Method of Evaluation for the Bone Mineral Density (BMD) and Area of Evaluation	Bone Mineral Density (Adjusted)			Study Conclusions Related to Bone Structure, Bone Metabolism and BMD
	M to F Transwomen	F to M Transmen		M to F	F to M			Before OR Controls	After OR Test	
**Schlatterer K et al. 1998** [37] **Berlin, Germany**	10	10	11.5 years	Parenteral high doses of estrogens + Cyproterone Acetate or Estrogens alone (2–8 mg/day)	Parenteral testosterone esters (250 mg every 2–4 weeks)	Conventional whole-body CT scanner	M to F	195 ± 20 mgr/ccm	174 ± 3 mgr/ccm	Slight reduction in the BMD of M to F M to F and F to M transsexuals treated with the proposed cross-gender hormone concept possess low risk of osteoporotic change
							F to M	174 ± 3 mgr/ccm	172 ± 2 mgr/ccm	
**Sosa M et al. 2003** [38] **Canary Islands, Spain**	53	-	201 ± 108 months or (16.75 ± 9) years	Ethinyl oestradiol + ciproterone acetate, -Oral estrogens and oestradiol valerate Specific dosages were not provided	-	Densitometer BMD of the lumbar spine and femoral neck	M to F	Lumbar Spine 1.002 ± 0.155 (gr/cm^2^)	Lumbar Spine 1.091 ± 0.150 (gr/cm^2^)	The chronic administration of estrogens in men may produce an increase in serum estradiol, a decrease in free testosterone levels, and an increase in BMD—Both in lumbar spine and in femoral neck. This study suggests that the bone of adult men is sensitive to estrogens.
								Femoral Neck 0.808 ± 0.135 (gr/cm^2^)	Femoral Neck 0.904 ± 0.135 (gr/cm^2^)	
**Ruetsche AG et al. 2005** [39] **Berne, Switzerland**	24	15	12.5 years M-F 2.1 years before surgery and 9.7 years after surgery F-M 1.3 Years before surgery and 6.1 years after surgery	Cyproterone acetate 2 mg/day + Ethinyl estradiol 35 µg/day or a free combination of Cyproterone acetate 2 mg/day + Ethinyl estradiol 35–100 µg/day After surgery, Estradiol valerate or Micronized 17-beta estradiol 2–4 mg/day.	Parenteral testosterone esters 250 mg/IM every 3 weeks Continued after surgery	Dual-energy X-ray absorptiometry (DXA) BMD of the lumbar spine, femoral neck, whole body, distal epiphysis, and other 5 areas	M to F	Lumbar spine 1.078 ± 0.131 (gr/cm^2^)	Lumbar spine 1.056 ± 0.137 (gr/cm^2^)	Biochemical values of calcium phosphate metabolism parameters were within normal ranges and comparable across groups. In transsexual genetic males and females under long term cross-sex hormone treatment, BMD values are generally preserved or increased. Non-compliance with cross-sex hormone treatment may lead to low BMD, only in genetic males. IGF-1 (Insulin like growth factor) could play a role in the mediation of the effect of androgens on bone in F-M transsexuals.
								Femoral neck 0.835 ± 0.100 (gr/cm^2^)	Femoral Neck 0.774 ± 0.095 (gr/cm^2^)	
								-	Whole body 1.216 ± 0.098 (gr/cm^2^)	
							F to M	Lumbar spine 1.100 ± 0.139 (gr/cm^2^)	Lumbar spine 1.075 ± 0.088 (gr/cm^2^)	
								Femoral neck 0.937 ± 0.121 (gr/cm^2^)	Femoral Neck 0.842 ± 0.058 (gr/cm^2^)	
								-	Whole body 1.179 ± 0.035 (gr/cm^2^)	
**Lapauw B et al. 2008** [40] **Ghent, Belgium**	23	-	>3 years At least 3 years under hormone treatment. Al the patients had sex reassignment surgery	Before surgery Cyproterone acetate 50–100 mg/day + ethinyl estradiol 25–50 μg/day After surgery either: -Ethinyl estradiol 25–50 μg/day (8 participants) -Estradiol valerate 2 mg/day (10 participants) -Conjugated equine estrogens1.25 mg/day (2 participants) -Transdermal estradiol gels (3 persons)	-	Dual-energy X-ray absorptiometry (DXA) BMD at the lumbar spine, distal forearm and Peripheral quantitative computed tomography (pQCT), at the same areas for analysis of the bone architecture	M to F	Lumbar spine 1.05 ± 0.11 (gr/cm^2^)	Lumbar spine 0.92 ± 0.14 (gr/cm^2^)	M to F transsexual persons presents: Lower muscle mass and strength and higher fat mass Lower trabecular bone density and BMD at various sites and smaller cortical bone size as compared to healthy age- and height-matched controls. The lower level of sports-related physical activity as well as the pharmacological and surgical withdrawal from endogenous T production could contribute to these findings. Male-to-female transsexuals may be at increased risk for developing osteoporosis and related fractures. Bone health should be a parameter of interest in the long-term follow-up care for male-to-female transsexual persons.
								Distal forearm 0.49 ± 0.05 (gr/cm^2^)	Distal forearm 0.42 ± 0.07 (gr/cm^2^)	
**T’Sjoen G et al. 2009** [41] **Ghent, Belgium**	50	-	>3 years and at least 1 year after sex reassignment	Cyproterone acetate 50–100 mg/day (1 year) + exogenous estrogen administration	-	Dual-energy X-ray absorptiometry (DXA) BMD at the lumbar spine, proximal femur and the distal radius of the nondominant site AND pQCT for the analysis of bone architecture	M to F	-	Lumbar spine 0.959 ± 0.159	Low bone mass, smaller bone size, and reduced muscle mass. Are highly prevalent in the described group of M-F transsexual persons Androgen deficiency or an inadequate estrogen dosage could be the cause Hormonal protocols differ between different centers and individual changes in BMD are highly variable There is a need for longitudinal single- and multi-center data on low bone mass risk in the M/F transsexual group.
									Total hip 0.940 ± 0.150	
									Radius and 0.432 ± 0.077	
**Van Caenegem, et al. 2012** [42] **Ghent, Belgium**	-	66 50 undergone surgery and received hormone therapy 16 Just hormone therapy	8.7 years after sex reassignment surgery (SRS) with a minimum of 9 months and a maximum of 22 years.	-	Testosterone decanoate 100 mg, Testosterone isocaproate OR fenylpropionate 60 mg, Testosterone propionate 30 mg/mL; 2–3 weeks (35 patients); Testosterone undecanoate 1000 mg; 12 weeks (7 patients); Transdermal testosterone 50 mg daily (8 patients) Testosterone undecanoate 40 mg/day + testosterone gel 50 mg per 5 g, 50 mg daily (1 Patient)	Dual-energy X-ray absorptiometry (DXA); BMD at the lumbar spine, and left proximal femur (total hip and femoral neck region)	F to M	Lumbar spine 1.06 ± 0.11 (gr/cm^2^)	Lumbar spine 1.03 ± 0.10 (gr/cm^2^)	Transmen (F-M) with hormone treatment and after SRS possess a bone and body composition comparable to men, compared with age-matched female controls. This is less fat mass, more central pattern of adiposity, more muscle mass, strength, and larger cortical bone size. The differences may result from the effects of long-term testosterone administration and of diminished estrogen exposure and/or from indirect effects through muscle mass and strength. Transsexual men (F to M) on long-term hormonal therapy do not have an increased risk of low bone mass but associated cardiovascular risk factors are important to address.
								Femoral neck 0.84 ± 0.10 (gr/cm^2^)	Femoral neck 0.82 ± 0.11 (gr/cm^2^)	
								Total hip 0.95 ± 0.10 (gr/cm^2^)	Total hip 0.96 ± 0.12 (gr/cm^2^)	
**Wierckx K et al. 2012** [43] **Ghent, Belgium**	50	50	±10 years	Before surgery Cyproterone acetate 50–100 mg/day/1 year different +exogenous estrogen After surgery all received estrogens (3 patients did not followed the estrogen protocol)	Testosterone	Dual-energy X-ray absorptiometry (DXA); BMD at the lumbar spine, at the proximal femur (total hip region), and a both distal forearms	M to F	Data not shown	Data not shown	After an average of 10 years of hormone treatment no important side effects were reported and osteoporosis was not observed in transsexual men (F to M). Transsexual women (M to F) suffered from osteoporosis at the lumbar spine and distal arm. Twelve percent of transsexual women (M to F) experienced thromboembolic and/or other cardiovascular events during hormone treatment, possibly related to older age, estrogen treatment, and lifestyle factors. In order to decrease cardiovascular morbidity, more attention should be paid to decrease cardiovascular risk factors during hormone therapy management.
							F to M	Data not shown	Data not shown	
**Wiepjes C et al. 2018** [44] **Amsterdam, Netherland**	711	543	10 years	Oral or transdermal estrogens and anti-androgens until gonadectomy	Oral, transdermal, or intramuscular testosterone.	Dual-energy X-ray absorptiometry (DEXA) at 2, 5, and 10 years Absolute BMD	M to F	Male and Female adult reference population	0.956 (+0.006) (gr/cm^2^)	This study showed that hormone therapy does not negatively affect the BMD Regularly assessing BMD should be completed just when osteoporotic risk is present (>60 years age) High percentage of low BMD was found prior to hormone therapy in transwomen. Therefore, evaluation of BMD before start of hormone therapy may be considered.
							F to M	Male and Female adult reference population	1.45 (+0.008) (gr/cm^2^)	
**Broulik PD et al. 2018** [45] **Prague, Czech Republic**	-	35	18 years	-	Before surgery Testosterone isobutyrate Sex reassignment surgery (hysterectomy, ovariectomy, and bilateral mastectomy) After surgery Testosterone isobutyrate 25 mg intramuscular every week, OR Testosterone propionate 250 mg every third week intramuscular OR Testosterone undecanoate 40 mg 4 times day.	Dual-energy X-ray absorptiometry (DXA); BMD at the lumbar spine and femoral neck	F to M	Male controls	Lumbar spine 1.213 ± 0.15	BMD after adequate dose of testosterone therapy is higher after 18 years of testosterone administration BMD at the spine its similar to baseline after 18 years of testosterone administration. Androgens compensate for the low estrogen level in the bone metabolism
								Lumbar spine 1.203 ± 0.065		
								Femoral neck 1.192 ± 0.19		
								Female controls	Femoral neck 0.950 ± 0.11	
								Lumbar spine 1.192 ± 0.19		
								Femoral neck 0.822 ± 0.09		
Summary of Findings	M to F 921	F to M 719	Time Receiving the Treatment (Range) >3 years to 18 years	M to F Hormone Therapy Cyproterone Acetate (Antiandrogen) Estrogens	F to M Hormone Therapy Testosterone	BMD Method of Evaluation Conventional whole-body scanner; Dual-energy X-ray absorptiometry (DXA)	-	-	BMD Contradictory results, the BMD was preserved or increased in 788 M to F patients (82.66%); BMD decreased in 73 M to F patients (8.47%); BMD Increased in 35 F to M patients (4.93%); BMD preserved in 674 F to M patients (95.06%)	

**Table 2:** Bone metabolism markers were reported in three of the included studies. No differences were observed before and after long-term pharmacotherapy for transmen or transwomen.

Author and Year of Publication	Sample		Bone Metabolism Markers								
	M to F	F to M	Calcium (mmol/L) or (mg/dL)			Phosphate (mmol/L)		Alkaline phosphatase (U/L)		Osteocalcin (µg/L) or (ng/mL)	
				Before or Controls	After or Tests	Before or Controls	After or Tests	Before or Controls	After or Tests	Before or Controls	After or Tests
Schlatterer K et al. 1998 [37]	10	10	M to F	Not evaluated	Not evaluated	Not evaluated	Not evaluated	Not evaluated	Not evaluated	Not evaluated	Not evaluated
			F to M	Not evaluated	Not evaluated	Not evaluated	Not evaluated	Not evaluated	Not evaluated		
Sosa M et al. 2003 [38]	53	-	M to F	9.4 ± 0.520 mg/dL	9.156 ± 0.564 mg/dL	3.348 ± 0.457 mg/dL	3.16 ± 0.619 (mg/dL)	Not evaluated	Not evaluated	Not evaluated	Not evaluated
Ruetsche AG et al. 2005 [39]	24	15	M to F	2.10–2.55 mmol/L	2.33 ± 0.08 (2.18–2.53) mmol/L	0.74–1.55 mmol/L	1.15 ± 0.12 (0.76–1.49) (mmol/L)	36–108 (μkat/L)	63 ± 15 (32–159) (μkat/L)	2.3–13.8 (ng/mL)	5.0 ± 1.0 (2.3–9.1) (ng/mL)
			F to M	2.10–2.55 mmol/L	2.38 ± 0.02 (2.32–2.52) mmol/L	0.74–1.55 mmol/L	1.05 ± 0.08 (0.69–1.23) (mmol/L)	36–120 (μkat/L)	6.3 ± 1.5 (3.4–11.4) (μkat/L)	1.2–10.5 (ng/mL)	6.3 ± 1.5 (3.4–11.4) (ng/mL)
Lapauw B et al. 2008 [40]	23	-	M to F	Not evaluated	Not evaluated	Not evaluated	Not evaluated	Not evaluated	Not evaluated	Not evaluated	Not evaluated
T’Sjoen G et al. 2009 [41]	50		M to F	Not evaluated	Not evaluated	Not evaluated	Not evaluated	Not evaluated	Not evaluated	Not evaluated	Not evaluated
Van Caenegem, et al. 2012 [42]	-	66	F to M	Not evaluated	Not evaluated	Not evaluated	Not evaluated	Not evaluated	Not evaluated	Not evaluated	Not evaluated
Wierckx K et al. 2012 [43]	50	50	T to F	Not evaluated	Not evaluated	Not evaluated	Not evaluated	Not evaluated	Not evaluated	Not evaluated	Not evaluated
			F to M	Not evaluated	Not evaluated	Not evaluated	Not evaluated	Not evaluated	Not evaluated	Not evaluated	Not evaluated
Wiepjes C et al. 2018 [44]	711	543	M to F	Not evaluated	Not evaluated	Not evaluated	Not evaluated	Not evaluated	Not evaluated	Not evaluated	Not evaluated
			F to M	Not evaluated	Not evaluated	Not evaluated	Not evaluated	Not evaluated	Not evaluated	Not evaluated	Not evaluated
Broulik PD et al. 2018 [45]	-	35	F to M	Not evaluated	Not evaluated	Not evaluated	Not evaluated	Female controls 1.51 ± 0.10 (μkat/L)	1.48 ± 0.12 (μkat/L)	Female controls 24.05 ± 6.8 (µg/L)	22.04 ± 7.92 (µg/L)
								Male controls 1.39 ± 0.14 (μkat/L)		Male controls 23.5 ± 8.0 (µg/L)	

M to F: A reduction of calcium of −0.244 (mg/dL), and reduction of phosphate of −0.188 (mg/dL) in the patients receiving hormone therapy compared to controls was observed by Sosa et al. 2003 [38]. No differences in calcium, phosphate, alkaline phosphatase, or osteocalcin in the patients receiving hormone therapy compared to controls by Ruetsche et al. 2005 [39]. F to M: No differences in calcium or phosphate were observed. Reduction in alkaline phosphatase from 36–120 (Ukat/L) in controls compared to 6.3 ± 1.5 (3.4–11.4) in tests. No changes in osteocalcin in the patients receiving hormone therapy compared to controls [39]. No changes were found in alkaline phosphatase and osteocalcin in patients receiving hormone therapy compared to male and female controls [45].

**Table 3:** Bone turnover markers were evaluated in five of the articles. Variable results were obtained for Serum Procollagen type I N-Terminal propeptide (P1NP) and for Serum collagen type I crosslinked C-telopeptide (CTX). P1NP increased in transwomen and transmen. CTX showed similar values before and after treatment.

Author and Year of Publication	Sample		Bone Turnover Markers				
	M to F	F to M	Serum Procollagen Type I N-Terminal Propeptide (P1NP) Formation (ng/mL)			Serum Collagen type I Crosslinked C-Telopeptide (CTX) Resorption (ng/mL)	
				Before or Controls	After or Tests	Before or Controls	After or Tests
Schlatterer K et al. 1998 [37]	10	10	M to F	M to F	Not evaluated	Not evaluated	Not evaluated
			F to M	F to M	Not evaluated	Not evaluated	Not evaluated
Sosa M et al. 2003 [38]	53	-	M to F	Not evaluated	Not evaluated	Not evaluated	Not evaluated
Ruetsche AG et al. 2005 [39]	24	15	M to F	Not evaluated	Not evaluated	Not evaluated	Not evaluated
			F to M	Not evaluated	Not evaluated	Not evaluated	Not evaluated
Lapauw B et al. 2008 [40]	23	-	M to F	32 [24,25,26,27,28,29,30,31,32,33,34,35,36,37,38,39,40,41,42,43,44,45] (46 controls)	49 [36,37,38,39,40,41,42,43,44,45,46,47,48,49,50,51,52,53,54,55,56,57,58,59,60,61,62] (23 patients)	0.36 ± 0.16 (46 controls)	0.24 ± 0.14 (23 patients)
T’Sjoen G et al. 2009 [41]	50	-	M to F	Lumbar spine 36.6 ± 22.6	Lumbar spine 45.2 ± 24.4	0.31 ± 0.20	0.32 ± 0.23
Van Caenegem, et al. 2012 [42]	-	66	F to M Before Surgery	40 ± 12	50 ± 24	0.20 ± 0.10	0.36 ± 0.15
Wierckx K et al. 2012 [43]	50	50	M to F	102 ng/mL	106–125 (ng/mL) (2 patients, all the others were within normal ranges)	<0.58 (ng/dL)	0.62–1.24 (ng/dL) (4 patients, all the others were within normal ranges)
			F to M	Normal range	Normal range	Normal range	Normal range
Wiepjes C et al. 2018 [44]	711	543	M to F	Not evaluated	Not evaluated	Not evaluated	Not evaluated
			F to M	Not evaluated	Not evaluated	Not evaluated	Not evaluated
Broulik PD et al. 2018 [45]	-	35	F to M	Not evaluated	Not evaluated	Control Female 400 ± 124	302 ± 190
						Control Male 390 ± 140	

PINP in M to F: Bone formation marker PINP increased from 32–49 ng/mL [40], and from 102–125 ng/mL [43]. PINP in F to M: Bone formation marker PINP increased from 40 ± 12–50 ± 24 ng/mL [42]. CTX in M to F: Bone resorption marker, decreased in one study from 0.36 ± 0.16–0.24 ± 0.14 ng/mL [40], and increased in another study from <0.58 ng/dL to 0.62–1.24 ng/dL [43]. CTX in F to M: Bone resorption marker CTX increased from 0.20 ± 0.10–0.36 ± 0.15 ng/mL [42], and was maintained within the normal range [41,43], and decreased from 400 ± 124–302 ± 190 ng/mL [45].
